# Characteristics of Inpatient Falls not Reported in an Incident Reporting System

**DOI:** 10.5539/gjhs.v8n3p17

**Published:** 2015-06-25

**Authors:** Shin-ichi Toyabe

**Affiliations:** 1 Niigata University Crisis Management Office, Niigata University Hospital, Niigata, Japan

**Keywords:** falls, adverse events, incident reports, text mining, natural language processing

## Abstract

An incident reporting system is the most commonly used method to identify patient safety incidents in a hospital. However, non-reporting of incidents for various reasons is a serious problem. We studied the rate of inpatient falls that were not reported in an incident reporting system but were recorded in medical charts and we evaluated characteristics of those falls by comparing with the falls reported in incident reports in a Japanese acute care hospital setting. Falls recorded in medical charts were detected by using a text mining method followed by a manual chart review. About 25% of the recorded falls were not reported in incident reports. Male patients, first fall, long lag time until recording, no witness at the time of the fall and physician profession were shown to be significant factors associated with non-reporting. Our results show that the rate of non-reporting of inpatient falls in a Japanese acute care hospital is compable to that shown in previous studies in other conutries and that the same barriers to incident reporting as those found in previous studies exist in the medical staff.

## 1. Introduction

An incident reporting system is commonly used to identify patient safety incidents or adverse events in a hospital (Aspden et al., 2004). However, non-reporting is an inevitable problem in this method because the method relies on voluntary willingness of medical staff (Cullen et al., 1995; Oliver et al., 2007). In addition, a significant lag time between incidents and submission of incident reports impairs quick detection of incidents (Hirose et al., 2007). The non-reporting of incidents is especially problematic when the incidents result in serious or fatal injuries. In addition, this problem might affect the results of epidemiological studies on incidents, planning of effective countermeasures to reduce the incidents, and evaluation of the countermeasures taken against the incidents.

Inpatient falls are the most common incidents that occur in a hospital. Since about 3 to 10% of inpatient falls in a hospital result in physical injuries including bone fracture and intracranial hemorrhage, it is necessary to identify injurious falls quickly (Toyabe, 2010, 2012a, 2012b). One strategy to prevent inpatient falls is a targeted intervention that focuses on patients at high risk for falls (Gates, Fisher, Cooke, Carter, & Lamb, 2008). Therefore, accurate epidemiology of falls in a hospital is necessary to determine whether the intervention for high-risk patients is effective. The non-reporting problem might prevent quick detection of severe cases and hinder evaluation of the effectiveness of intervention against inpatient falls.

It has been suggested in previous reports that there are various barriers to incident reporting that lead to non-reporting (Evans, Cameron, Myles, Stoelwinder, & McNeil, 2005; Waring, 2005). However, there are only few reports about factors that affect non-reporting of inpatient falls (Hill et al., 2010) and there is no report of this problem in a Japanese acute care hospital setting. The aim of this study was to clarify the rate of inpatient falls that are not reported by an incident reporting system and what factors affect the non-reporting of inpatient falls in a Japanese acute care hospital setting. To answer the questions, we analyzed significant characteristics of falls that were not reported in incident reports but were recorded in medical charts, as compared with falls that were reported in incident reports. We performed a two-step procedure of the first screening with a text mining method and then confirmed the results of the first screening by a manual chart review.

## 2. Methods

### 2.1 Setting

This study was conducted at Niigata University Hospital, an 825-bed academic teaching hospital in the city of Niigata. There are 23 clinical departments and the service area of the hospital as a tertiary care hospital covers all districts in Niigata Prefecture, which has a population of 2 400 000. All patients who had been admitted to the hospital during the period from June 2011 and August 2011 were studied. During the period, 4439 patients (52 551 patient-days) were admitted to the hospital.

### 2.2 Data Collection

Information on patients’ background including age, gender, cognitive status, major diagnostic category of the patient's principal diagnosis, admission ward, admission day and discharge day was obtained from the hospital information system (HIS). Information on falls recorded by incident reports was obtained from online incident reporting system. Incident reports contained information on degree of injury of the patient, type of event and essential information on the event such as the name of the patient, the name of the medical staff involved, the exact time and place that the event occurred, detailed information on the course of the incident, action taken by medical staff and outcome of the event. In addition to the data on falls obtained from incident reports, data on falls were also obtained from progress notes of the electronic medical record (EMR) by using a text mining method (Toyabe, 2012a). Briefly, the text data of progress notes of the EMR were obtained from the HIS electronically. One unit of record corresponds to text data written at a time in progress notes by medical staff. The progress notes were written by various types of staff including physicians, nurses, and other medical staff. The text data were then applied to morphological analysis, which is a process of segmenting a sentence into a row of morphemes. The row of morphemes was applied to syntactic analysis to determine the grammatical structure of the sentence and the dependency relationship between the morphemes. These pretreated data were then analyzed to determine whether they contained sets of morphemes and their dependency relationship that were specific for the text data in which the occurrence of the fall events was described. Text mining analyses were performed using the software Text Mining Studio (NTT DATA Mathematical Systems Inc., Tokyo, Japan). The sensitivity of the text mining analysis to detect fall events from the progress notes was as high as 100%, but the positive predictive value was as low as 6% (Toyabe, 2012a). Therefore, the sentences detected by text mining analysis were checked by a manual chart review to determine whether they really contained information on the occurrence of fall events.

### 2.3 Statistical Analyses and Ethical Consideration

Fall events detected from incident reports and progress notes of the EMR were divided into three groups in terms of where the falls events were recorded: falls recorded only in incident reports, falls recorded only in progress notes, and falls recorded in both incident reports and progress notes. These three categories of fall events were compared in terms of factors that could influence the recording or reporting of fall events such as patient background and fall-related information. Comparison of discrete variables between the groups was performed using the chi-square test and Fisher's exact test. Continuous variables such as lag time between falls and data submission were expressed as medians (25th-percentile, 75th-percentile), and comparison of unpaired continuous variables between groups was performed by Kruskal-Wallis’ test and Wilcoxon's rank sum test. A paired comparison of groups was performed by using Friedman's test or Wilcoxon's signed rank test. Multivariate logistic analysis was performed to find factors that were associated with non-reporting among the above-mentioned various factors. A stepwise selection method was used to determine the most significant factors. In all statistical analyses, a p-value less than 0.05 was considered significant. All statistical analyses except text mining analysis were performed by using SPSS Statistics 22 (IBM Japan Ltd., Tokyo, Japan). This study was approved by the Ethics Committee of Niigata University School of Medicine.

## 3. Results

### 3.1 Falls Detected From Medical Records

From progress notes of the EMR for the patients, we electronically obtained 640 434 records. These data were subjected to text mining analysis, and records suspected to have fall-related information were obtained (Table 1). A total of 10 200 records (1.59%) were suspected to have fall-related information among the 640 434 records. The fall-related information could be divided into five categories which consisted of fall motion (slipping or tripping), losing balance of the body, injuries suffered by falls, falling from the bed, and use of a fall-detecting sensor for patients at risk for falls. The most frequent category was losing balance of the body (5567, 54.58%), followed by fall motion (4297, 42.13%). The 10,200 records obtained by text mining were then checked by a manual chart review. As a result, 635 records (6.23%) out of the 10 200 records actually contained information on falls. The other 9565 records that were initially suspected to have fall-related information were finally found to have no information on actual falls. They contained information related to risk assessment of falls, falls that could be avoided before they happened, or falls that occurred before the patients were admitted to our hospital. Among the five categories of fall-related information, the category that contained the largest number of records was that related to fall motion (555 out of 635, 87.40%). On the other hand, the category related to injuries suffered from falls contained information on actual falls most frequently (89 out of 320, 27.81%). Eventually, 635 records (0.10%) related to 164 actual fall events were obtained from 640 434 records from 52 551 patient-days. Therefore, the rate of recorded falls in our hospital was calculated to be 3.12 per 1,000 patient-days.

**Table 1:** Results of text mining and subsequent chart review of progress notes of the EMR to detect inpatient falls

Fall-related information	Number of records suspected to have fall-related information	Number of records that contained information on true fall events	Rate of true fall events
			
Fall motion (slips or trips)	4297	555	12.92%
Losing balance of the body	5567	66	1.19%
Injury suffered from falls	320	89	27.81%
Drop from bed	381	48	12.60%
Use of fall-detecting sensor	342	6	1.75%

Total	10 200	635	6.23%

### 3.2 Types of Record of Falls

**Figure 1:**
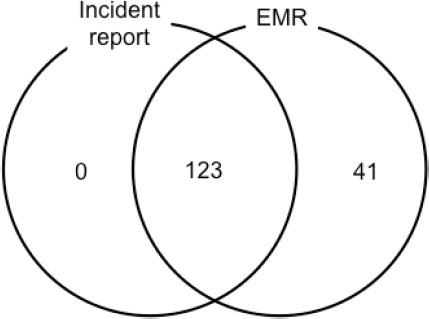
Fall events captured from incident reports and progress notes of the EMR

Among the 164 fall events, 123 falls (75.0%) were reported in incident reports. The other 41 falls (25.0%) were recorded in progress notes of the EMR but were not reported in incident reports. There were no falls that were reported in incident reports but were not recorded in the EMR (Fig. 1). We initially planned to focus on three groups for comparison, but the comparison was made between two groups: falls that were recorded both in the EMR and incident reports and falls that were recorded in the EMR but were not reported in incident reports.

### 3.3 Characteristics of Falls not Reported in the Incident Reporting System

Various characteristics of falls reported in incident reports and falls not reported in incident reports were compared (Table 2). Falls not reported in incident reports were more likely to include falls of male patients (p<0.001), first falls (p=0.001), falls that occurred on a holiday (p=0.024), falls that occurred without a witness (p<0.001) and falls recorded by physicians (p<0.001). On the other hand, falls not recorded in incident reports were less likely to include falls of patients with a cognitive disorder (p=0.002), falls that occurred during the night shift (p=0.019), injurious falls (p=0.041) and falls recorded by nurses (p<0.001). Lag time between falls occurring and submission of the data into the EMR was significantly longer for falls not reported by incident reports than for falls reported by incident reports (p=0.002). When the lag time was analyzed for falls that were recorded in both incident reports and progress notes of the EMR, the lag time in incident reports was significantly longer than the lag time in progress notes of the EMR (p<0.001). Multivariate logistic analysis with a stepwise selection method was performed to determine factors that were most significantly associated with falls not reported by incident reports (Table 3). Male gender, first fall, long lag time between occurrence of the fall and submission of data, no witness at the time of the fall and falls recorded by physicians were significantly associated with falls not recorded in incident reports.

**Table 2:** Comparison of the characteristics of falls reported in incident reports and those of falls not reported in incident reports

		Total falls		Falls recorded in incident reports		Falls not recorded in incident reports		Sig.
N		164		123		41		
Age		66.5 (51.0, 78.0)		67.0 (51.0, 79.0)		62.0 (49.0, 74.0)		0.163
Male		42 (25.6%)		14 (11.4%)		28 (68.3%)		<0.001
Cognitive disorder		28 (17.1%)		27 (22.0%)		1 (2.4%)		0.002
Major diagnostic category								0.156
Nervous system		22 (13.4%)		20 (16.3%)		2 (4.9%)		
Ear, nose, mouth and throat		12 (7.3%)		5 (4.1%)		7 (17.1%)		
Respiratory system		15 (9.1%)		9 (7.3%)		6 (14.6%)		
Circulatory system		10 (6.1%)		6 (4.9%)		4 (9.8%)		
Digestive system		27 (16.5%)		19 (15.4%)		8 (19.5%)		
Hepatobiliary system and pancreas		15 (9.1%)		11 (8.9%)		4 (9.8%)		
Kidney and urinary tract		16 (9.8%)		12 (9.8%)		4 (9.8%)		
Male reproductive system		14 (8.5%)		13 (10.6%)		1 (2.4%)		
Myeloproliverative diseases		14 (8.5%)		11 (8.9%)		3 (7.3%)		
Others		19 (11.6%)		17 (13.8%)		2 (4.9%)		
Ward								0.088
A		12 (7.3%)		6 (4.9%)		6 (14.6%)		
B		18 (11.0%)		17 (13.8%)		1 (2.4%)		
C		12 (7.3%)		8 (6.5%)		4 (9.8%)		
D		14 (8.5%)		11 (8.9%)		3 (7.3%)		
E		13 (7.9%)		10 (8.1%)		3 (7.3%)		
F		12 (7.3%)		8 (6.5%)		4 (9.8%)		
G		17 (10.4%)		10 (8.1%)		7 (17.1%)		
Others		66 (40.2%)		52 (42.3%)		11 (26.8%)		
				
First fall		107 (65.2%)		72 (58.5%)		35 (85.4%)		0.001	
Falls that occurred on a holiday		39 (23.8%)		24 (19.5%)		15 (36.6%)		0.024	
Time of falls (shift)								0.037	
8:30 to 16:30 (day)		69 (42.1%)		47 (38.2%)		22 (53.7%)		0.123	
16:30 to 24:00 (twilight)		45 (27.4%)		32 (26.0%)		13 (31.7%)		0.613	
0:00 to 8:30 (night)		50 (30.5%)		44 (35.8%)		6 (14.6%)		0.019	
No witness at the time of a fall		53 (32.3%)		21 (17.1%)		32 (78.0%)		<0.001	
Location of fall								0.557	
Out of room		42 (25.6%)		29 (23.6%)		13 (31.7%)			
Bedside		62 (37.8%)		47 (38.2%)		15 (36.6%)			
Diagnostic imaging		19 (11.6%)		17 (13.8%)		2 (4.9%)		0.097	
Injurious falls		32 (19.5%)		29 (23.6%)		3 (7.3%)		0.041	
Mild		26 (15.9%)		24 (19.5%)		2 (4.9%)		0.048	
Moderate to severe		6 (3.7%)		5 (4.1%)		1 (2.4%)		1.000	
Person who recorded fall								<0.001	
Physician		16 (9.8%)		2 (1.6%)		14 (34.1%)		<0.001	
Nurses		142 (86.6%)		116 (94.3%)		26 (63.4%)		<0.001	
Others		6 (3.7%)		5 (4.1%)		1 (2.4%)		1.000	
Lag time between episode and record		2.0 (0.0, 4.0)		1.0 (0.0, 4.0)		4.0 (1.0, 8.0)		0.002	

**Table 3:** Characteristics of falls not reported in incident reports

	B	S.E.	p-value	Exp (B)	95% C.I. for Exp (B)	
					Lower	Upper
Male gender	3.302	0.765	<0.001	27.167	6.070	121.584
First fall	2.154	0.931	0.021	8.617	1.390	53.438
Lag time	0.226	0.085	0.008	1.254	1.061	1.482
Witness	-2.709	0.694	<0.001	0.067	0.017	0.260
Person who found fall (vs nurses)			0.002			
Physicians	4.487	1.270	<0.001	88.869	7.379	1070.238
Others	-17.076	18 590.892	0.999	0.000	0.000	
Constant	-3.785	1.066	<0.001	0.023		

A total of 640,434 records obtained from progress notes of the EMR were subjected to text mining analysis, and 10,200 records (1.59%) were suspected to have fall-related information. The fall-related information could be divided into five categories. The records that were captured by text mining analysis were subsequently examined by a manual chart review. Eventually, 635 true fall events were identified (0.10% of total records). Since a record suspected to have fall events and a record that has information on true fall events could belong to more than one of the categories of fall-related information, the sum of number of records belonging to each category was not equal to the total number of records.

Various characteristics of falls reported in incident reports and falls not reported in incident reports were compared.

Multivariate logistic analysis with a stepwise variable selection method was used to find factors that were significantly associated with non-reporting.

## 4. Discussion

Our results showed that 25% of recorded falls were not reported in the incident reporting system. It is well known that a voluntary incident reporting system can detect only a part of the incidents occurring in a hospital (Sari, Sheldon, Cracknell, & Turnbull, 2007). This situation is the same for inpatient falls. Healey et al. reported that the rate of falls in acute hospitals varied remarkably between hospitals from 0.2 to 11.5 (average 4.8) falls per 1,000 bed days based on incident reports and that this variability in the rate of falls was mainly due to reporting bias of medical staff (Healey et al., 2008). Hill et al. reported that hospital incident reporting systems captured only 75.5% of fall events (Hill et al., 2010), and Grenier-Sennelier et al. reported that 20.4% of inpatient falls were not reported in incident reports (Grenier-Sennelier, Lombard, Jeny-Loeper, Maillet-Gouret & Minvielle, 2002). These figures of non-reporting of inpatient falls are comparable to our results. Our results show that the non-reporting problem in inpatient falls is also the same in a Japanese acute care hospital setting.

The non-reporting problem is especially important when the precise incidence and detailed information on incidents are required. Examples of such situations include epidemiological study of inpatient falls, validation of countermeasures against falls, and development of risk assessment systems for inpatient falls. In reality, fall incidence in our hospital was estimated to be 2.34 per 1,000 bed days based only on information from incident reports, but it was calculated to be 3.12 per 1,000 bed days based on the results of a chart review. There is a remarkable difference, which might lead to an incorrect conclusion that the present countermeasures against inpatient falls are effective.

This non-reporting problem occurs with the background of various barriers to incident reporting. Previous studies showed that uncertainness of which incidents and why the incidents should be reported, cumbersome procedure to report, lack of feedback to reporters, and culture of blame in the organization were inhibiting factors for incident reporting (Evans et al., 2005). We tried to clarify the barriers to incident reporting in inpatient falls. Our results showed that male patients, initial falls, falls that were not found immediately by medical staff, falls found by physicians, and falls that took a long time until medical staff knew the events were significant factors for medical staff not reporting the events in the incident reporting system. The reason why falls of male patients were less likely to be reported in the incident reporting system is unknown. One possible reason is that male patients might be less likely to sustain an injury from falling (Hitcho et al., 2004), and medical staff were shown to be more likely to report injurious falls (Hill et al., 2010). In accordance with this speculation regarding the reason, we observed that injurious falls were more likely to be reported in the incident reporting system in our study. Falls that were not noticed immediately by medical staff or falls that took a long time until medical staff became aware of them were less likely to be reported in the incident reporting system. Patients who experinence falls often do not inform medical staff about their falls, and medical staff often become aware that a patient has fallen by chance from conversation with the patient. Since these falls are apparently not injurious, they seem less likely to be reported in the incident reporting system. Other factors that were found to influnece non-reporting in this study were in accordance with the results of the previous studies. The first fall was less likely to be reported in the incident reporting system as was shown in previous studies (Hill et al., 2010). Falls found by physicians were less likely to be reported in the incident reporting system. It is well known that physicians report incident reports less frequently than do nurses (Evans et al., 2005). The attitude towards incidents and participation in the incident reporting system varied between physicians and other medical professionals (Waring, 2004).

In order to deal with the non-reporting problem, use of more than one method to detect medical incidents is recommended (Olsen et al., 2007; Naessens et al., 2009). Since incidents identified by one method were not identified using another method (Naessens et al., 2009), combination use of several methods to detect incidents instead of the use of a single method is important. At present, information on medical incidents mainly originates from a retrospective chart review (Olsen et al., 2007) and voluntary reports from health care professionals (Medicine 2004). However, several other methods have been used to identify these events. Hospital patients’ reports revealed a number of incidents that were not recorded in medical records (Davis, Sevdalis, Neale, Massey, & Vincent, 2012). A full-time fall evaluation service consisting of trained nurses could remarkably increase the capture of fall events (Shorr et al., 2008). The Agency for Healthcare Research and Quality (AHRQ)-defined patient safety indicators (PSI) using International Classification of Diseases (ICD) - diagnosis codes from discharge abstracts can detect a larger number of incidents than an incident reporting system can (Naessens et al., 2009). Recently, medical record reviewing by using the Institute for Healthcare Improvement (IHI) Global Trigger Tool has become increasingly popular because it could identify a much larger extent than that detected by an incident reporting system (Classen et al., 2011; Rutberg et al., 2014). The Global Trigger Tool uses specific methods for reviewing medical charts by several staff that are trained to review the charts in a systemic manner (Classen et al., 2011). Although the methods can detect a greater amount of incidents, staff education and the chart reviewing process could be time-consuming and costly.

In the present study, we used an information technology-assisted retrospective chart review. We used a text mining method in the first screening process of the chart review, and the screened records were manually confirmed to actually contain information on falls. By using this method, we could reduce candidates for manual chart review to 1.6% of the original text data. As medical information becomes increasingly computerized, automated methods to detect incidents using information technology have been developed (Govindan, Van Citters, Nelson, Kelly-Cummings, & Suresh, 2010). Detection of incidents using computerized methods to scan medical charts requires less time and personnel resources than those used in traditional methods. On the other hand, the text-mining method or natural language processing method has a number of limitations and shortcomings for daily use. First, the method cannot detect adverse events that were not recorded in any fields of medical charts and can only find adverse events recorded in an object data field. For example, radiology reports have been used for detection of patient falls in previous studies (Bates et al., 2003). However, we found that the efficiency for detection of fall events from radiology reports or image order entries was very low compared with that from progress notes (Toyabe, 2012a). Diagnostic imaging is not often performed in patients who seem not to have suffered injuries after falls. Second, false-positive results are difficult to avoid in the method. Our method was not simple keyword searching but was a method for capturing context corresponding to inpatient falls from sentences. Nevertheless, the positive predictive value of our method was quite low in some of the fall-related information categories. The development of a more sophisticated algorithm in those categories is needed for improvement of the false positive rate and for practical use of the method.

## 5. Conclusion

Our results showed that 25% of recorded falls were not reported in the incident reporting system and that the rate of non-reporting of inpatient falls in a Japanese acute care hospital is compable to that found in previous studies in other conutries. Non-reporting of fall events was significantly associated with several factors including male patients, first fall, long lag time until recording, no witness at the time of the fall and physician profession. The results suggest that the barriers to incident reporting that exist in the medical staff of a Japanese acute care hospital are the same as those found in previous studies in other countries.
